# *Moringa oleifera* leaves extract-mediated synthesis of ZnO nanostructures for the enhanced photocatalytic oxidation of erythrosine†

**DOI:** 10.1039/d4ra08782h

**Published:** 2025-01-27

**Authors:** Noshaba Parven, Khalida Faryal Almani, Muhammad Ali Bhatti, Aneela Tahira, Omm-e-Hany, Aqeel Ahmed Shah, Ayman Nafady, Matteo Tonezzer, Zafar Hussain Ibupoto

**Affiliations:** a Departement of Pharmaceuticals, University of Sindh Jamshoro 76080 Pakistan; b Centre for Environmental Sciences, University of Sindh Jamshoro Sindh 76080 Pakistan; c Institute of Chemistry, University of Sindh Jamshoro 76080 Pakistan zaffar.ibhupoto@usindh.edu.pk; d Institute of Chemistry, Shah Abdul Latif University Khairpur Mirs Sindh Pakistan; e Department of Environmental Studies, University of Karachi Sindh 75270 Pakistan; f Wet Chemistry Laboratory, Department of Metallurgical Engineering, NED University of Engineering and Technology University Road Karachi 75270 Pakistan; g Chemistry Department, College of Science, King Saud University Riyadh 11451 Saudi Arabia; h Department of Chemical and Geological Sciences, University of Cagliari Monserrato Italy

## Abstract

This study was focused on the development of ZnO nanostructures for the efficient oxidation of erythrosine dye and for studying the antibacterial activity of ZnO. It was observed that the phytochemicals from *Moringa oleifera* leaves modified the size, shape, crystalline properties and surface chemical composition of the ZnO nanostructures. ZnO nanostructures synthesized with 15 mL *Moringa oleifera* leaves extract (S-15) demonstrated highly efficient oxidation of erythrosine dye under the illumination of natural sunlight. Various photocatalyst evaluation parameters, such as initial dye concentration, pH of the dye solution, catalyst dose and cycling stability, were studied. The S-15 sample of ZnO exhibited almost 100% dye removal in an alkaline pH of 12 and a low concentration of 4.54 × 10^−5^ M. Furthermore, improved antibacterial activity was also observed against *E. coli* and *Bacillus subtilis* bacteria strains. The use of *Moringa oleifera* leaves extract could be considered a low-cost, facile and ecofriendly green synthesis protocol for replacing the use of toxic chemicals and for eliminating the risk of releasing of toxic chemicals into the environment during the synthesis of high-performance nanostructured materials.

## Abstract 2

This study was focused on the development of ZnO nanostructures for the efficient oxidation of erythrosine dye and for studying the antibacterial activity of ZnO.

## Graphical abstract

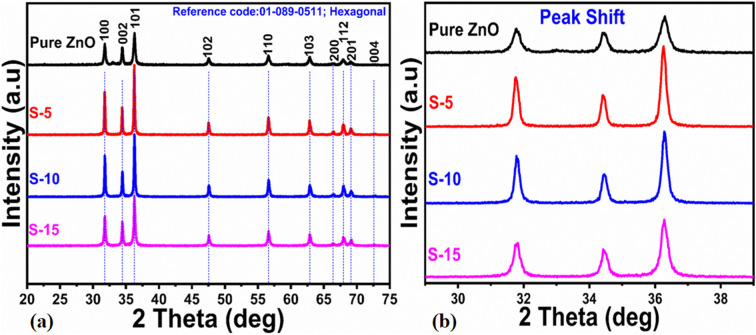

## 1. Introduction

Water scarcity is a global problem, and therefore, it has attracted significant technological and scientific interests.^1^ The rise in the industries and their enormous product formation rate has steeply increased owing to the global increase in the human population. Consequently, there are tens of millions of synthetic dyes that are employed as coloring agents for the clothing, food, pharmaceuticals and textile industries, which are now posing major concerns in the form of wastewater pollution.^2,3^ Among the dyes, erythrosine is a well-known water soluble dye, and it is commonly named as Red No. 3. It is among the class of xanthene dyes. The exposure of erythrosine *via* industrial wastewater can cause several health issues to the human body.^4,5^ For instance, it was reported that erythrosine dye can cause thyroid issues because it contains iodine in its molecular structure, which may become exposed to the human body during degradation. Hence, erythrosine can be responsible for atopic illnesses.^6^ It is also highly carcinogenic and can cause tumors.^7,8^ Alongside these adverse effects of erythrosine, a low dose of its exposure can result in stomach, liver, kidney colon, lung, urinary bladder, brain, and DNA damage and bone marrow issues.^9^ Erythrosine dye causes neurotoxicity *via* a disorder in the neurotransmitter uptake under *in vitro* conditions.^10,11^ The xenoestrogen stimulates estrogens.^12,13^ These negative aspects of erythrosine dye highlight the urgent need to remove it from wastewater using systematic and efficient technologies. Furthermore, given the coloring and complicated molecular structure of dyes, they are not easily removable from wastewater using routine wastewater treatment methods. Hence, the removal of dyes from industrial effluents is considered a major problem for industrialists and researchers. Different potential methods have been employed for the removal of dyes from the industrial effluents. Additionally, wastewater typically carries different types of bacteria along with the dye, which can cause contamination in water bodies and have highly adverse effects on our lives. It has been reported that bacteria and dyes can cause a significant pollution of water bodies, which can even kill the aquatic life therein, especially fish and aquatic flora.^14,15^ Furthermore, the consumption of contaminated water by animals and humans can lead to different health risks, such as respiratory and neurological disorders, and skin issues. Along with the adsorption techniques used for the removal of colored dyes, chemical oxidation is particularly useful, with hydrogen peroxide, chlorine, coagulation, settling, and filtration commonly used.^16,17^ Currently, erythrosine dye is mainly removed by adsorption methods.^18,19^ However, adsorption and chemical oxidation methods have limitations, including the very costly fabrication of efficient adsorbents, and the high cost of chemicals used in chemical oxidation for the removal of dyes from the wastewater.^20^ Thus, research has been performed to develop new, low-cost materials, and sustainable methods for the efficient removal of dyes from industrial effluents.^21^ Photocatalysis is one of the main advanced oxidation processes investigated for the low cost and efficient removal of dyes. Hence, it is preferred over existing dye removal methods due to its high efficiency, no generation of secondary pollutants, inexpensiveness, and ecofriendly nature. This is the reason why photocatalytic oxidation is attracting relatively more interest than conventional methods for the degradation of organic pollutants, especially dyes form wastewater.^22,23^ Generally, the photocatalytic oxidation of organic pollutants takes place *via* semiconducting nanoparticles under the irradiation of sunlight in aqueous solution. The degradation of dyes arises through the combined relation of the energy of sunlight photons and the band gap of semiconductor materials under the irradiation of sunlight. During sunlight irradiation, the influence of photons with a relatively higher energy than the band gap energy of a material can result in the valence electrons receiving enough energy to move up to the conduction band, leaving behind holes in the valence band. Hence, the holes promote the reduction process while electrons promote the oxidation process, which is actually the reason for the degradation of dyes under photocatalytic oxidation.^24–27^ For these reasons, various nanostructured photocatalysts, including Ag, Ag_3_PO_4_, CdS, CuO, V_2_O_5_, TiO_2_, g-C_3_N_4_, SiO_2,_ ZnO, porphyrin, AB_2_X_4_, and their composites systems, have been found to be highly efficient photocatalytic materials for the removal of organic dyes from wastewater.^28–33^ In particular, ZnO has been extensively studied owing to its good catalytic activity and significant photosensitivity for creating electron–hole pairs during interaction with photons of sufficient energy.^34^ Also, ZnO exhibits significant antibacterial activity. These features of ZnO enable it to be used as an efficient photocatalytic and as part of an antibacterial protocol for the degradation of organic dyes for removing wastewater contamination, thereby playing a vital role in strengthening the sustainability of the environment. ZnO also exhibits high photostability, indicating its long-term sustainable activity under continuous sunlight irradiation.^35^ Despite an intensive growth in the reports on the synthesis of a wide range of ZnO nanostructures through different methods for photocatalytic and antibacterial applications, the performance ZnO is still inadequate for use in practical applications. Therefore, new strategies with a facile, low cost, and ecofriendly nature are required to enhance the performance of ZnO nanostructures. The photocatalytic oxidation and the antibacterial activity of nanostructured materials highly depend on their synthetic route, size, shape, and surface chemical composition. From the perspective of material design, green synthesis has received increasingly intensive attention among researchers owing to its facile and ecofriendly nature. In the green synthesis of nanostructured materials, different parts of plants, such as the seed, leaf, stem, aerial parts, bark peels, flowers, and fruit rinds, have been used. These plant parts have chemical compositions including phytochemicals, such as alkaloids, phenols, tannins, phenols, saponins, alkaloids, steroids, and cardiac glycosides.^36–38^ However, few studies of *Moringa oleifera* leaves extract and its phytochemicals analysis have been carried out, although these phytochemicals have been used for the surface modification of ZnO nanostructures and their antibacterial activities have been reported.^39–44^ In these reported works, the effect of *Moringa oleifera* leaves extract was evaluated considering the structure, optical, and antibacterial activities of materials; however, the role of different amounts of *Moringa oleifera* leaves extract used in the synthesis of nanostructures for optimizing their photocatalytic activities has rarely been studied. The previous studies suggest there is plenty of room to use *Moringa oleifera* leaves extract for the enhanced photocatalytic oxidation and antibacterial activities. There are also few reports on the photocatalytic oxidation of erythrosine dye. Therefore, we report for the first time the role of different amounts of *Moringa oleifera* leaves extract in modifying the surface properties of ZnO nanostructures towards the oxidation of erythrosine dye. Beside the photodegradation of erythrosine dye, an enhanced antibacterial activity was also observed.

## 2. Experimental section

### 2.1. Chemical reagents

Zinc acetate dihydrate Zn(CH_3_CO_2_)_2_ (≥99.0%), aqueous ammonia solution NH_4_OH (≥98%), ethanol C_2_H_6_O (≥99.9%), and methylene blue (99%) were purchased from Sigma-Aldrich Karachi, Sindh, Pakistan. Solutions with specific concentrations were made using distilled water for use in the following experiments. *Moringa* plant leaves were picked from the Center for Environmental Sciences, University of Sindh, and Jamshoro, Pakistan.

### 2.2. Preparing *Moringa oleifera* leaves extract and synthesis of ZnO nanostructures

**Scheme 1:**
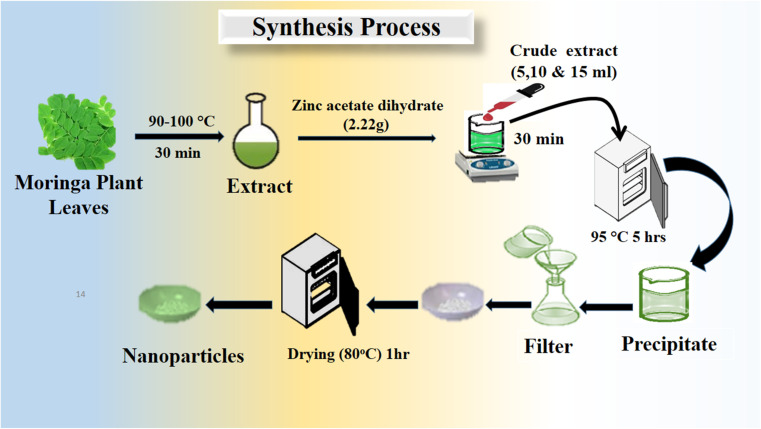
Stepwise preparation of various ZnO nanostructures, including S-5, S-10 and S-15, using *Moringa oleifera* leaves extract.

*Moringa oleifera* plant leaves were picked from a live planted tree, and then washed with distilled water for removing the dirt from the leaves and consequently dried at 60 °C in an oven. Later, a pestle and mortar were used to grind the dried leaves. Then, 10 g *Moringa oleifera* plant leaves powder was combined with 100 mL distilled water in a glass beaker. Finally, the extract was obtained by heating at 80–85 °C for 30 min, and then allowed to cool at room temperature. The *Moringa oleifera* leaves extract was stored in a refrigerator at 4 °C when not in use. The growth solutions were prepared using 2.22 g of zinc acetate dihydrate Zn(CH_3_CO_2_) in 95 mL distilled water, and then 5 mL ammonia solution was added drop-wise for a total volume growth solution of about 100 mL. This growth solution was labeled as pure ZnO. For the exploitation of useful phytochemicals from the *Moringa oleifera* leaves extract for the synthesis of ZnO nanostructures, three sets of growth solution beakers were taken, containing 5, 10, and 15 mL, respectively, of *Moringa oleifera* leaves extract in addition to 22.2 g zinc acetate dihydrate. Then, 5 mL aqueous ammonia solution was added in to each growth solution beaker followed by the addition of distilled water for a total volume of solution of 100 mL. The growth solutions with 5, 10, and 15 mL *Moringa oleifera* leaves extract were named as S-5, S-10, and S-15, respectively. The hydrothermal process was performed on these growth solutions, which were tightly covered with an aluminum sheet for 5 h at 95 °C. Finally, the ZnO precipitates were collected and dried at 80 °C for 1 h. A brief illustration of the growth process is presented in Scheme 1.

### 2.3. Physical characterization

The morphologies of the pure ZnO and *Moringa oleifera* leaves extract-assisted ZnO samples were studied by scanning electron microscopy (ESEM FEG Philips) at 20 kV. Energy-dispersive X-ray (EDX) was used for the elemental analysis. Powder X-ray diffraction (XRD) was employed to record the diffraction patterns of the different ZnO samples under the conditions: Cu Kα radiation, 40 kV, 40 mA, with a step of 0.03° (2*θ*), and a scanning speed of 0.06° s^−1^. Infrared experiments were done by Fourier transform infrared (FTIR) spectroscopy (Thermo Scientific Nicolet 95 iS10, Waltham, MA, USA) in the frequency range of 4000–450 cm^−1^. A UV-visible spectrophotometer (Shimadzu UV-2600i) was employed to evaluate the optical band gap and the degradation of erythrosine dye in water under natural sunlight irradiation.

### 2.4. Photocatalytic studies of the *Moringa oleifera* leaves extract-mediated ZnO nanostructures

Photocatalytic degradation of erythrosine dye was done under the illumination of natural sunlight according to the following process: a 50 mL solution with two different concentrations of 9.09 × 10^−5^ M and 4.54 × 10^−5^ M of dye, containing 10 mg catalyst, was used. Samples were taken out at different intervals of time and assessed *via* UV spectroscopy at a wavelength of 526 nm to quantify the remaining dye concentration in the solution. To determine the final degradation efficiency, the below equation was used1
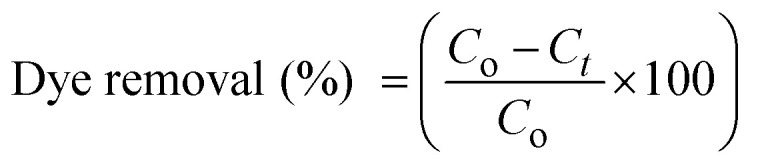
where *C*_*t*_ represents the dye concentration at time *t*, and *C*_0_ is the initial concentration.

The degradation kinetics was studied using the following relation: 
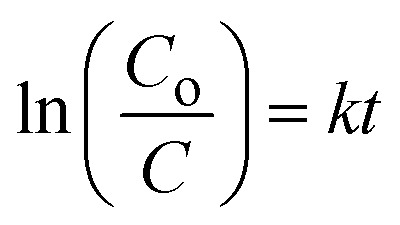
, where *C*_0_ is the initial erythrosine concentration, *C* is the residual erythrosine concentration at different illumination intervals, *k* (min^−1^) is the pseudo-first-order rate kinetic constant, and *t* represents the irradiation time. The cycling stability of the most efficient sample was done in 4.54 × 10^5^ M dye solution with a catalyst dose of 15 mg, while the sample could be recovered from the used dye solution using centrifugation at an rpm of 5500, and it was repeated for 5 times. The degradation process was used as described above. The scavenger test was done using 20 mL of each scavenger in 4.54 × 10^−5^ M dye solution. The agar well diffusion method was employed for the evaluation of the antibacterial activity of different as-synthesized ZnO nanostructures and tested against *E. coli* and *Bacillus subtilis* bacteria strains. The microorganism were treated for 24 h at 37 °C, using saline solution for producing sufficient colony forming units. Later, the microbes were put on the surface of a Petri dish with a 5 mm crock borer. Then, the dilute solution was placed at 5 m and the inhibition zone was measured after 24 h. The measurements were repeated 3 times for achieving accurate antibacterial performance of the as-synthesized ZnO nanostructures.

## 3. Results and discussion

### 3.1. Structure, morphology, elemental, and optical studies of the *Moringa oleifera* leaves extract-mediated ZnO nanostructures

**Fig. 1:**
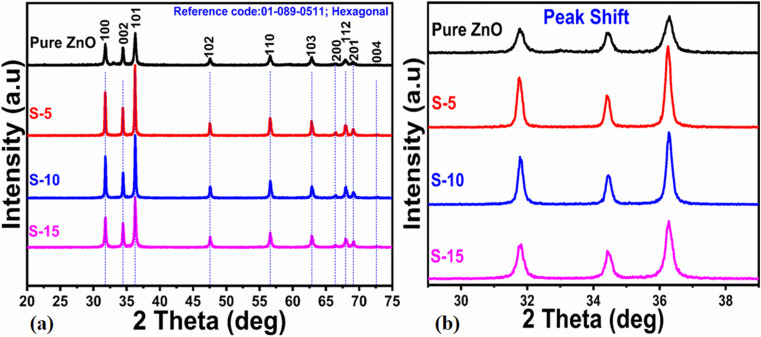
XRD patterns of (a) pure ZnO and different ZnO nanostructures, including S-5, S-10 and S-15, and (b) corresponding two theta shifts to higher angles for the extracts.

The atomic structure arrangement in the crystals of the as-synthesized ZnO nanostructures was analyzed by powder XRD. The diffraction patterns of various ZnO nanostructures were measured and are shown in Fig. 1. Using 5, 10, and 15 mL of *Moringa oleifera* leaves extract, we prepared three samples of ZnO, labeled as S-5 (5 mL), S-10 (10 mL), and S-15 (15 mL), and their diffraction patterns were compared with the ZnO nanostructures synthesized without *Moringa oleifera* leaves, extract as shown in Fig. 1a. All the ZnO samples exhibited the typical hexagonal phase and the measured diffraction patterns were in good agreement with the JCPDS card no: 01-089-0511. The Miller indices included (100), (002), (101), (102), (110), (103), (200), (112), (201), and (004), and could be clearly seen for all the samples, as shown in Fig. 1a. All these reflections fully satisfied JCDPS card no: 01-089-0511, confirming that the *Moringa oleifera* leaves extract did not induce any impurities in the ZnO nanostructures, and consequently high-purity ZnO crystals were obtained. Scherrer's formula was applied to estimate the average crystallite size of the ZnO nanostructures, with the Scherrer formula based on the *λ* (wavelength of X-rays), Bragg's angle, and *β* as the full width at half maximum of radium.^45^ For all the samples, the reflection peaks, such as (100), (002), and (101), were used for estimation of the average crystallite sizes of the as-synthesized ZnO. The average crystallite sizes of pure ZnO, S-5, S-10, and S-15 were estimated to be 77.0, 45.0, 44.0, and 35.0 nm, respectively, as shown in Table 1. This crystallite size analysis suggested that the different phytochemicals from the *Moringa oleifera* leaves extract, including phenolic and flavonoids compounds, significantly changed the nucleation rate of ZnO crystals, thus potentially impacting the crystallite sizes of the as-synthesized ZnO nanostructures. J. Vera *et al.*^46^ reported that the amount and nature of phenolic and flavonoid compounds from plant extracts can influence the nucleation rate and the size of ZnO nanoparticles. Moreover, the presence of different phenolic and flavonoid compounds has the possibility to offer different functional groups, like oxygenated atoms, which could bind with the zinc ions and form intermediate complexes during the growth process. Therefore, they can influence the growth kinetics and change the morphology of ZnO nanostructures. The sizes of the ZnO nanostructures could also be strongly influenced by the shape structure of the as-synthesized material. Extracts having high total phenolic and antioxidant contents have been reported to produce modified ZnO nanoparticles with smaller particle sizes.^46^ The *Moringa oleifera* leaves extract-mediated ZnO nanostructures possessed narrow and intense reflection peaks, indicating the synthesized materials had well-resolved crystalline properties and typical crystal structured.^47^ The XRD reflections of ZnO with *Moringa oleifera* leaves extract were similar to other reported green synthesis-fabricated ZnO nanostructures.^48,49^ Matinise *et al.*^50^ reported the synthesis of ZnO nanoparticles by employing *Moringa oleifera* leaves extract as an excellent Zn^2+^ chelating agent *via* its bioactive components. A significant change in the crystallite size was noticed. Surendra *et al.*^51^ prepared spherical ZnO nanoparticles from *Moringa oleifera* peels having crystallite sizes in the range of 40–45 nm. There was also a shift in two theta angle of ZnO nanostructures prepared with the addition of *Moringa oleifera* leaves extract, as shown in Fig. 1b, and this shift was larger when 15 mL *Moringa oleifera* leaves extract was used. The shift in the two theta angle could be related to the large phytochemical molecules playing a role during the nucleation stage of crystal growth, thereby causing stress on the overall synthesis of ZnO nanostructures.^48,49^

**Table 1:** Average crystalline size of the pure ZnO and green synthesized ZnO sample 1, sample 2 and sample 3 materials

Sample-ID	2 theta (deg.)	Peak position	FWHM	Height	Average crystalline size (nm)
Pure ZnO	100	31.78	0.1279	209	77
	002	34.4	0.06	188	
	101	36.3	0.29272	359	
Sample-1	100	31.76	0.18682	491	45
	002	34.4	0.17981	312	
	101	36.26	0.18288	802	
Sample-2	100	31.78	0.18204	473	44
	002	34.46	0.1924	283	
	101	36.28	0.19823	718	
Sample-3	100	31.82	0.23801	336	35
	002	34.42	0.23409	274	
	101	36.28	0.24522	567	

**Fig. 2:**
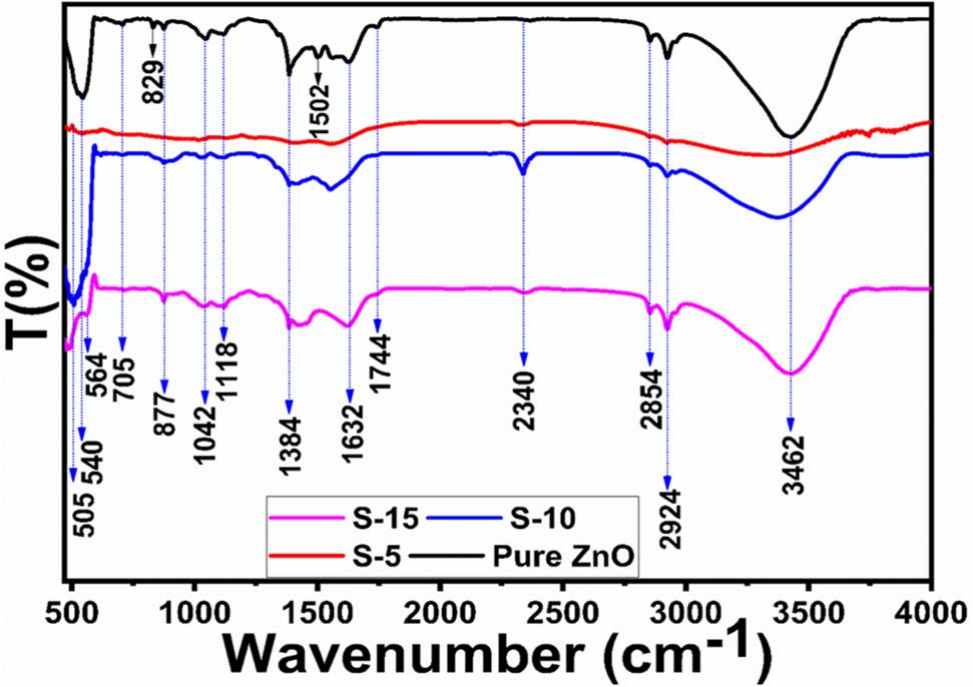
IR bands of pure ZnO and ZnO nanostructures, including S-5, S-10, and S-15, mediated with *Moringa oleifera* leaves extract.

To investigate the capping and stabilizing agents from the phytochemicals of *Moringa oleifera* leaves extract towards ZnO nanostructures, FTIR experiments were done. The typical FTIR spectra of pure ZnO, S-5, S-10, and S-15 are shown in Fig. 2. Typical bands of alkaloids, phenolic compounds, and flavonoids were noticed around 1632 (N–H) and (C–O) at 705 to 829 cm^−1^ or (RCOO) at 1384 cm^−1^.^52^ Also, the IR band in the range 1632–1744 cm^−1^ could be attributed to C=O stretching vibration for the flavonoid carbonyl groups. The hydroxyl groups from phenols and –NH_2_ stretching vibrations were associated with broad stretching bands at 3462 cm^−1^ in ZnO and could be attributed to the plant extract. The C–H stretching vibrations were noticed at 2924 and 2854 cm^−1^, while the N–H bending vibrations were noticed at 1502 cm^−1^. The bands between 1118–1384 cm^−1^ could belong to both C–H and C–C aromatic stretching modes for alkanes. The IR band at 1042 cm^−1^ indicated the presence of saturated primary alcohol C–O stretching vibrations. The presence of an absorption band at 2340 cm^−1^ emerged due to the aromatic aldehydes C–H IR stretching frequencies. The IR band at 1502 cm^−1^ could be indexed to carbonyl group originating from the flavonoids of the plant extract. The *Moringa oleifera* leaves extract-assisted ZnO nanostructures showed typical Zn–O stretching vibrations in the range of 500–564 cm^−1^.^53^ The phytochemicals from the *Moringa oleifera* leaves extract showed shifts in the position and intensity of its peaks during interaction with the ZnO.^54^ The FTIR results confirmed that the ZnO nanostructures were successfully formed and had different functional groups on their surface.

**Fig. 3:**
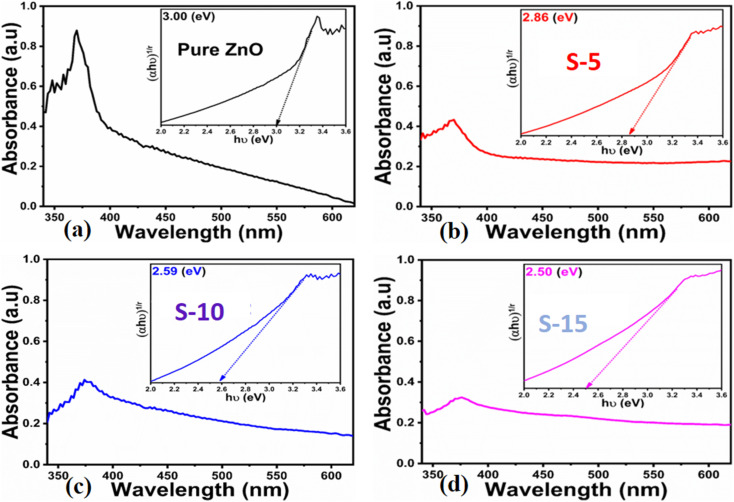
UV-visible absorption spectra of (a) pure ZnO, (b–d) ZnO nanostructures synthesized with *Moringa oleifera* leaves extract, such as S-5, S-10 and S-15; insets show the corresponding Tauc plots.

Next, optical characterization was carried out for pure ZnO, and for samples S-5, S-10, and S-15 of ZnO nanostructures obtained with *Moringa oleifera* leaves extract by using UV-visible absorption spectroscopy. The absorption spectra indicated the typical absorption band of ZnO around 380 nm because of electronic transition from the valence band to the conduction band, while the absorption band at 370 nm suggested the formation of ZnO compounds, as shown in Fig. 3a–d. There was a slight shift of the UV-visible absorption for S-15, which could be attributed to its modified size, shape, and surface properties. The optical band gap energy of ZnO could be obtained using a Tauc plot and from the following equation.^55^

2 (*αhv*)^2^ = *K*(*hv* − *E*_g_)^*n*^

where *K* is a constant, *hv* is the photon energy, *E*_g_ is the band gap energy, and *n* is an exponent.

**Fig. 4:**
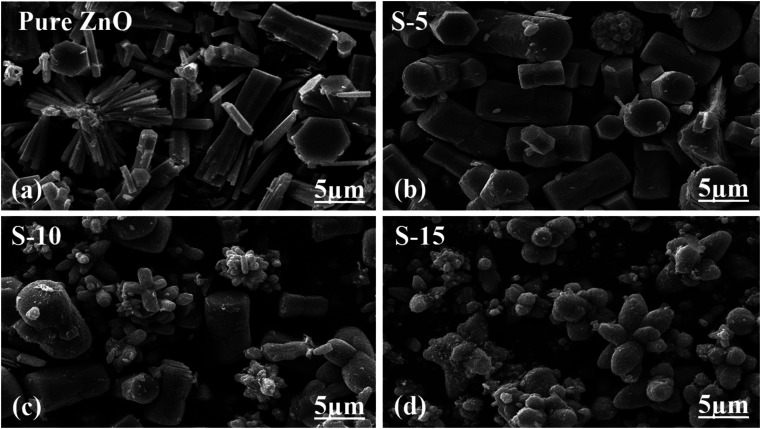
SEM images of (a) pure ZnO and (b–d) ZnO nanostructures synthesized with different amounts of *Moringa oleifera* leaves extract, such as S-5, S-10 and S-15.

The extrapolation of the linear part of the curves allows calculating the optical band gap of each ZnO sample, and their corresponding values are shown in the insets of Fig. 3a–d. The findings of the optical study revealed the optical band gaps of pure ZnO, S-5, S-10, and S-15 as 3.00, 2.86, 2.59, and 2.50 eV, respectively. These aspects of *Moringa oleifera* leaves extract-mediated ZnO indicate there was a reduced optical band gap, which could be attributed to the variation in the size, morphology, and crystal structure of ZnO. According to Yashni *et al.*,^56^ variations in the nature and quantity of phytochemicals from plant extracts may cause variances of the bandgap energy. The phytochemicals from *Moringa oleifera* leaves extract offered an abundant source of reducing, capping, and structure-directing agents for the modifying the surface, shape orientation, and size of ZnO nanostructures. Later, the variation in the size, and shape structure could change the electronic band structure of ZnO, thus a variation in the optical band gap could be noticed. The surface functional groups might also change the band energy *via* possible electron transfer to ZnO, and such aspects are being reported by other authors.^56^ Next, the shape features of ZnO nanostructures using *Moringa oleifera* leaves extract were studied by SEM and the typical SEM images of pure ZnO, S-5, S-10, and S-15 are shown in Fig. 4a–d. Pure ZnO displayed the typical nanorod-like morphology with obvious hexagonal facets, as shown in Fig. 4a. The diameters of the nanorods were around 500 nm and their lengths were several microns. The orientation of the nanorods in pure ZnO was irregular. The effect of *Moringa oleifera* leaves extract on the morphology could be very clearly observed and the effect became more prominent with increasing the volume of *Moringa oleifera* leaves extract during the synthesis of ZnO nanostructures, as shown in Fig. 4b and c. The addition of 5 mL of *Moringa oleifera* leaves extract during the growth slightly changed the hexagonal facets and the nanorods were shorter, as shown in Fig. 4b. The further addition of 10 mL *Moringa oleifera* leaves extract resulted in complete destruction of the nanorod structure into a heterostructure of ZnO, as shown in Fig. 4c. Also, the effect of *Moringa oleifera* leaves extract was more dynamic when 15 mL was used, whereby the nanorod structure was completely lost and changed into elongated cone-type particles and with a highly reduced size, as shown in Fig. 4d. As mentioned earlier, the phytochemicals of *Moringa oleifera* leaves extract include flavonoids, alkaloids, and phenolic compounds and glycosides with reducing, capping, stabilizing and structure-directing properties, hence small-sized ZnO structures were produced.^57^ The *Moringa oleifera* leaves extract-assisted ZnO nanostructures might have a different shape orientation with a large surface area because of their small dimensions, and thus a higher number of active sites could be expected.^58^ Furthermore, the generalized mechanism could be described as follows: the *Moringa oleifera* leaves extract contains flavonoids, phenolic acids, and tannins, which are associated with oxygenated atoms. These oxygenated atoms carry lone pairs, which can coordinate with the zinc ions during the growth process and form intermediate complexes, hence the nucleation rate of the ZnO crystal could be changed, and thereby an alteration in shape and size could be expected.

**Fig. 5:**
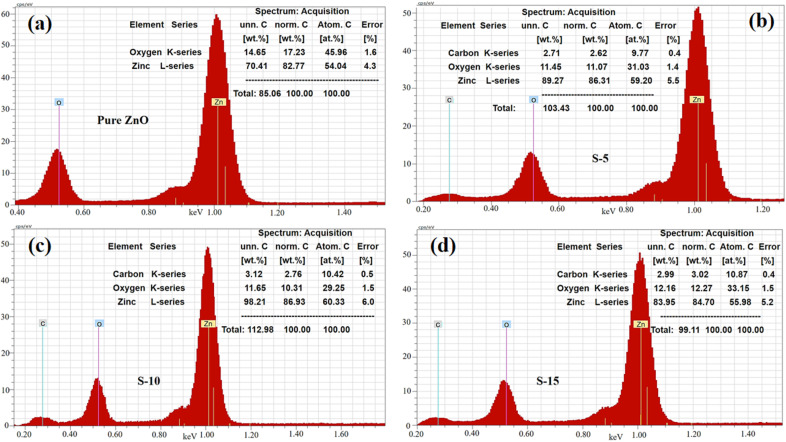
EDS spectra of (a) pure ZnO and (b–d) ZnO nanostructures synthesized with *Moringa oleifera* leaves extract, such as S-5, S-10, and S-15.

Next, EDX analysis was done on the pure ZnO, S-5, S-10, and S-15 prepared with *Moringa oleifera* leaves extract for identification of the elemental chemical composition, as shown in Fig. 5a–d. The corresponding EDX spectra are shown in Fig. 5a–d. All the EDX spectra confirmed the successful formation of ZnO and showed the samples only contained Zn and O as the main elements, while the C could be from the zinc precursors or *Moringa oleifera* leaves extract used during the synthesis of these samples, as shown in Fig. 5a–d. Each EDX spectrum was characterized with three signals and the length of each signal provided quantitative information of each element. Specifically, the three signals in the EDX spectra were associated with C, Zn, and O, as shown in Fig. 5a–d.

### 3.2. Photocatalytic oxidation of erythrosine using the *Moringa oleifera* leaves extract-mediated ZnO nanostructures

**Fig. 6:**
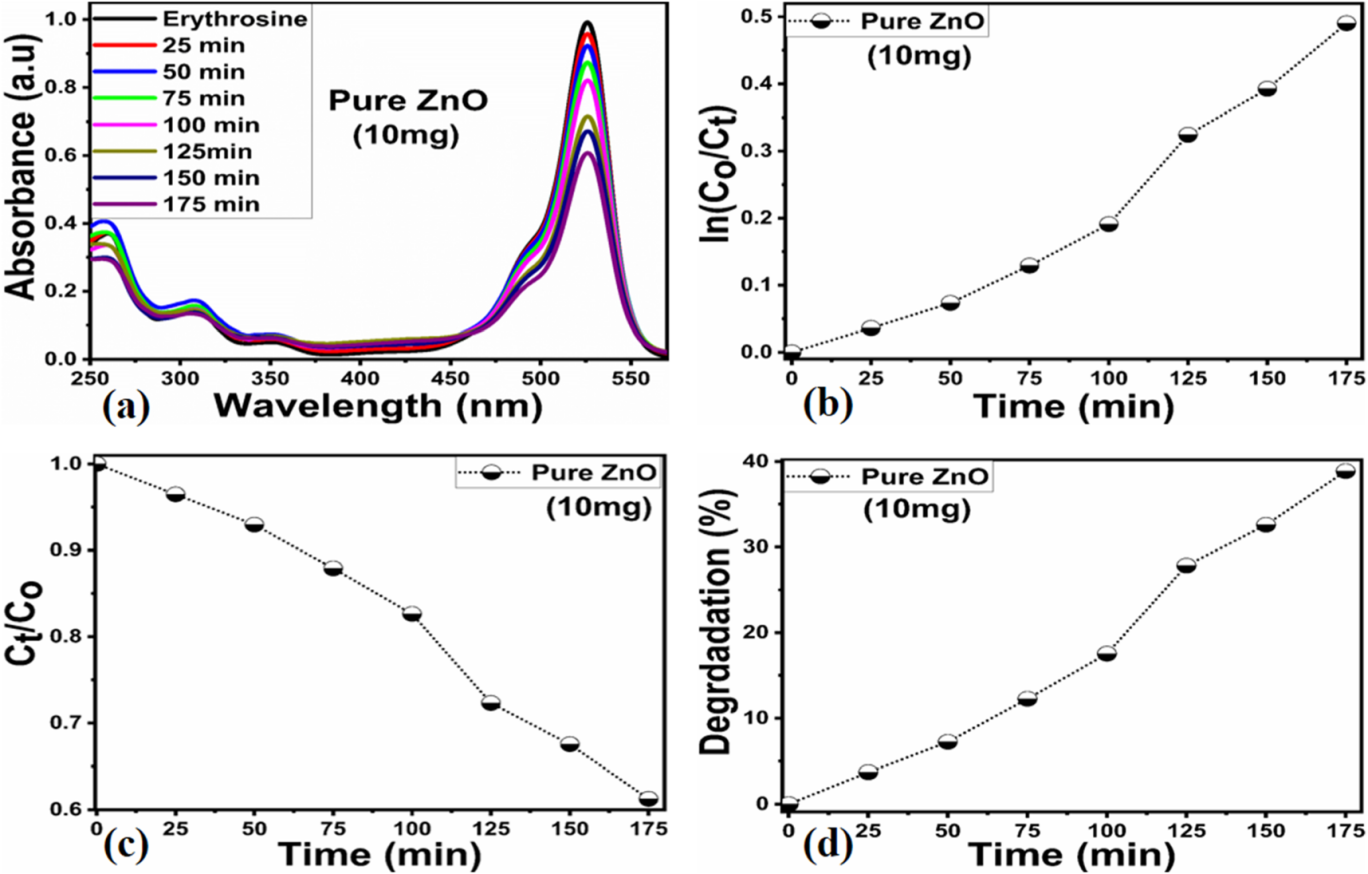
(a) UV-visible absorption spectra of erythrosine in the presence of pure ZnO, (b and c) degradation kinetics fitted with a pseudo-first-order model and the degradation rate, and (d) dye removal %.

**Fig. 7:**
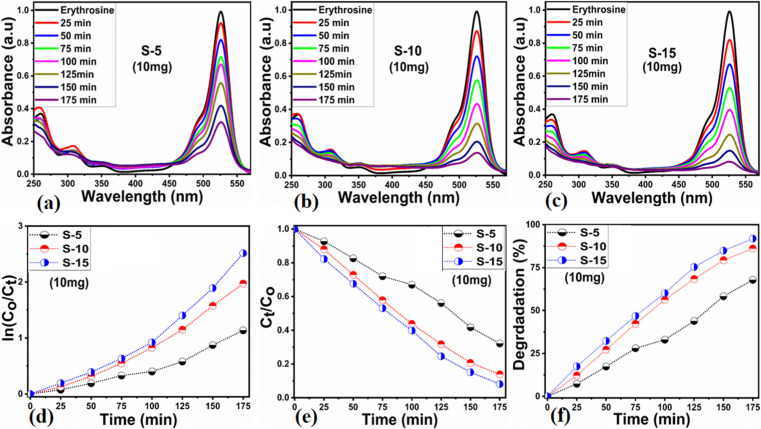
(a–c) UV-visible absorption spectra of erythrosine using ZnO nanostructures synthesized with *Moringa oleifera* leaves extract, such as S-5, S-10, and S-15, (d and e) corresponding degradation kinetics fitted with a pseudo-first-order model and the degradation rate, (f) observed dye removal % of S-5, S-10, and S-15.

Different sets of ZnO nanostructures were prepared with varying amounts of *Moringa oleifera* leaves extract and their performance was evaluated as photocatalytic materials against erythrosine under natural sunlight illumination. The photocatalytic performance of pure ZnO was compared with the *Moringa oleifera* leaves extract-assisted nanostructures. A catalyst dose of 10 mg pure ZnO was used in 9.09 × 10^−5^ M erythrosine prepared in water and was treated with natural sunlight, as shown in Fig. 6a. It could be observed that the relative decreases in the absorbance was very slow, indicating the poor catalytic activity of pure ZnO and also the stability of the erythrosine dye, hence the degradation rate was found to be highly limited. The UV-visible absorption was measured at 25 min intervals and the degradation of erythrosine was carried out for 175 min, as shown in Fig. 6a. The limiting catalytic activity of pure ZnO could be assigned to its wide band gap energy that could not produce enough electrons and holes during interaction with the natural sunlight photons. The fast recombination rate of electrons and holes could further worsen the photocatalytic performance of pure ZnO, thus new strategies are required to utilize ZnO-based photocatalysts for use in wastewater management. The erythrosine degradation rate constant was studied and it could be seen that the decomposition rate constant was very low, as indicated through the slope value of the fitting curve shown in Fig. 6b. The degradation rate of the erythrosine concentration was investigated and the relative degradation rate was found to be poor, suggesting the slow degradation rate of erythrosine dye, as shown in Fig. 6c. The dye removal % was estimated and the obtained results are shown in Fig. 6d. Again, a poor value of 37% degradation of erythrosine dye was observed. For these reasons, we performed a study into the oxidation of erythrosine dye using ZnO nanostructures prepared under the influence of 5 mL (S-5), 10 mL (S-10), and 15 mL (S-15) *Moringa oleifera* leaves extract, which showed an enhanced dye removal %. The different catalyst doses and 9.09 × 10^−5^ M erythrosine were employed under natural sunlight illumination, as shown in Fig. 7a–c. A reduction in the absorption of erythrosine was noticed after just 25 min, and the degradation was further monitored for a total period of 175 min. Interestingly, the decrease in the absorption was significant compared to that of pure ZnO, confirming the enhanced photocatalytic performance of ZnO nanostructures synthesized with different amounts of *Moringa oleifera* leaves extract, as shown in Fig. 7a. Among the biogenic ZnO samples, S-15 was found to be the most efficient in oxidizing erythrosine, as could be witnessed from its role in decreasing the absorption. The addition of *Moringa oleifera* leaves extract significantly modified the shape structure, surface properties, and optical features and minimized the recombination rate of electron–hole pairs, hence an excellent oxidation of erythrosine dye was demonstrated. When exposed to sunlight, electrons become excited and transfer from ZnO's valence band to an empty conduction band, leaving behind holes in the valence band, thus forming electron–hole pairs, which are thought to be good oxidizing and reducing agents. These electron–hole pairs interact with dissolved oxygen and water molecules and generate free radicals, which consequently decompose the dye in aqueous solution.^59^ Such favorable aspects from the modification in structure, size, optical band gap energy, and functional groups of ZnO nanostructures synthesized with *Moringa oleifera* leaves extract were observed *via* SEM, FTIR, XRD, and UV-visible spectroscopy. The degradation kinetics of erythrosine was also evaluated by employing the ZnO nanostructures (S-5, S-10, and S-15) prepared with different amounts of *Moringa oleifera* leaves extract. These fitting results are shown in Fig. 7b, where it could be seen that the corresponding slope values were relatively higher than that of pure ZnO, verifying the dynamic role of *Moringa oleifera* leaves extract in enhancing the catalytic performance of ZnO for the degradation of erythrosine. The degradation rate of erythrosine dye was also highly enhanced in the presence of biogenic ZnO nanostructures modified by *Moringa oleifera* leaves extract, as shown in Fig. 7c. It was also observed that the degradation rate of these modified ZnO samples was relatively faster than that of pure ZnO, which was related to the abundant catalytic sites in the modified materials. The dye removal % of ZnO samples was also determined and the corresponding dye removal performances of S-5, S-10, and S-15 are shown in Fig. 7f. The dye removal % of S-15 was higher than that of S-5 and S-10, although these were also better at removing erythrosine from aqueous solution compared to pure ZnO. The dye removal % of S-5, S-10, and S-15 were 67%, 86%, and 91%, respectively. These findings clearly show that *Moringa oleifera* leaves extract is a highly useful biomass offering exciting phytochemicals for improving the photocatalytic activity of ZnO towards the oxidation of erythrosine dye. Specifically, the *Moringa oleifera* leaves extract was an abundant source of phytochemicals that play a vital role in changing the size, shape, and crystal quality of ZnO nanostructures. Further, these aspects of *Moringa oleifera* leaves extract towards ZnO also affected the optical band gap of ZnO. The photocatalytic performance was strongly influenced by the shape structure and size of the nanostructured photocatalytic material, and we have observed that the size, shape, and modified surface properties of ZnO played vital roles in enhancing the photodegradation of erythrosine dye in aqueous solution.

**Fig. 8:**
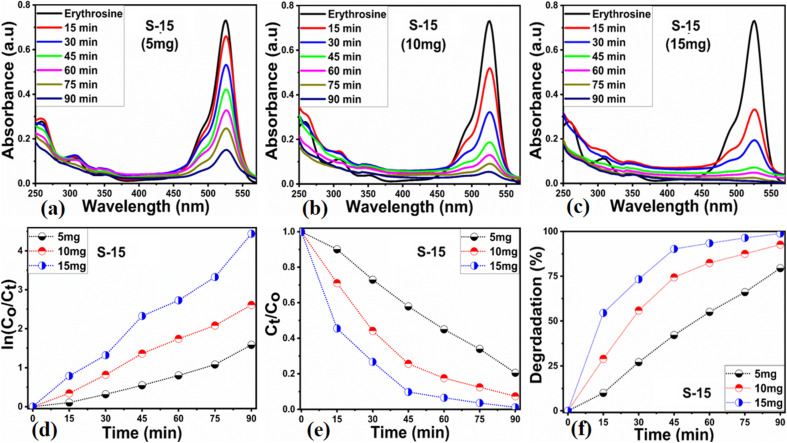
(a–c) UV-visible absorption spectra of erythrosine using ZnO nanostructures synthesized with *Moringa oleifera* leaves extract, namely, S-5, S-10, and S-15, and using catalyst doses of 5, 10 and 15 mg, (d and e) fitted pseudo-first-order model for the degradation kinetics and the degradation rate using 5, 10 and 15 mg catalyst doses, (f) dye removal % of S-15 using 5, 10, and 15 mg catalyst doses.

**Fig. 9:**
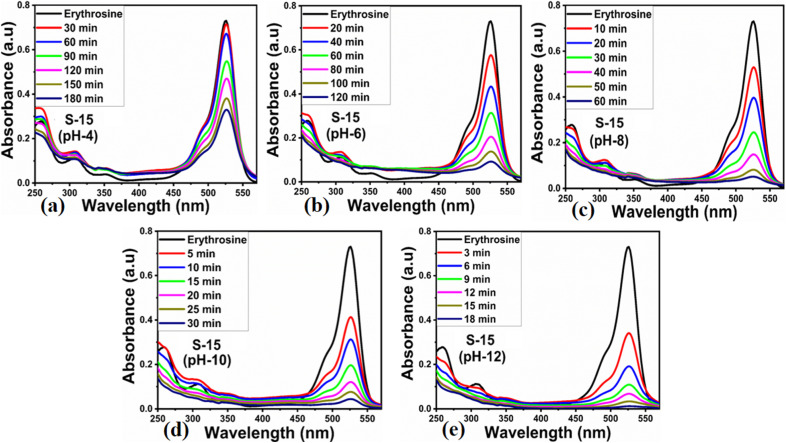
(a–e) UV-visible absorption spectra of erythrosine solution having pH value of 4, 6, 8, 10, and 12 using ZnO nanostructures synthesized with 15 mL *Moringa oleifera* leaves extract (S-15) using a catalyst dose of 15 mg.

**Fig. 10:**
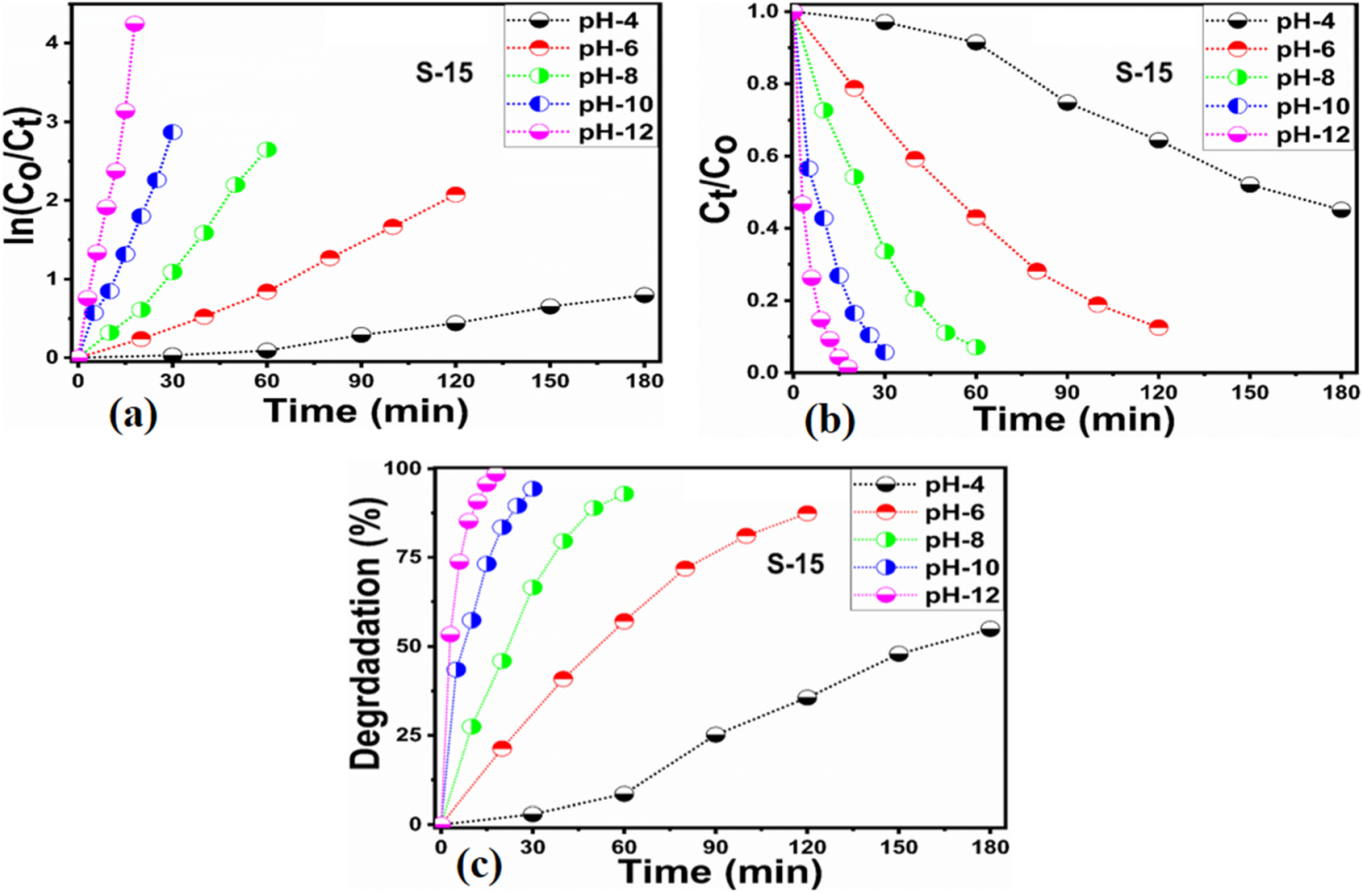
(a and b) Fitted pseudo-first-order model for the degradation kinetics and the degradation rate using 15 mg catalyst of ZnO nanostructures synthesized with 15 mL (S-15) *Moringa oleifera* leaves extract using a catalyst dose of 15 mg, (c) dye removal % of S-15 using a 15 mg catalyst dose.

Next, the initial dye concentration and catalyst dose parameters were explored using 5, 10, and 15 mg S-15 ZnO nanostructures synthesized with 15 mL *Moringa oleifera* leaves extract, while an erythrosine concentration of 4.54 × 10^−5^ M was used with each catalyst dose. The variation in the dye concentration at different time intervals was studied by observing the decrease in the absorbance during a UV-visible absorbance study, as shown in Fig. 8a–c. It could be seen that catalyst dose highly influenced the enhancement in the degradation of dye with increasing time. A higher catalyst dose also means a higher density of catalytic sites, hence the degradation rate could be accelerated at a rapid rate, as shown in Fig. 8a–c. Among the catalyst doses, 15 mg of S-15 was found to be highly efficient at increasing the dye degradation, as shown in Fig. 8c. A higher catalyst dose can adsorb more dye molecules and generate more electron–hole pairs, which can further offer a greater amount of oxidizing radicals through interaction with water and oxygen molecules in the dye solution, thus a rapid degradation of dye could be witnessed. The catalyst dose-dependent dye-degradation kinetics and rate were studied, as shown in Fig. 8d and e. It could be seen that the kinetics showed large rate constant values, with the higher rate of reaction significantly derived by the higher catalyst dose, as shown in Fig. 8d and e. The dye removal % was further evaluated for different catalyst doses of S-15, namely 5, 10, and 15 mg, as shown in Fig. 8f. The overall dye removal % results suggested that S-15 with 15 mg catalysts had the highest dye removal %, as shown in Fig. 8f, which could be assigned to its large surface area, high amount of catalytic sites, and abundant generation of electron–hole pairs for attaining a high density of oxidizing radicals. The effect of the pH of the erythrosine dye solution was monitored using 4.54 × 10^−5^ M with adjusting the pH values as 3, 6, 8, 10, and 12 and a catalyst dose of 15 mg S-15 of ZnO synthesized with *Moringa oleifera* leaves extract. The pH adjustment was done with 2 M HCl and NaOH aqueous solution. Later, all these adjusted conditions were irradiated with natural sunlight and the UV-visible absorbance spectra at different time intervals were collected and are shown in Fig. 9a–e. It was found that when using 15 mg S-15 of ZnO, varying the local pH environment of the photocatalyst could lead to different catalytic performances, as shown in Fig. 9a–e. The pH environment of the dye solution was associated with different amounts of hydroxyl ions, radicals, and catalyst tolerance under different pH values. This could be clearly seen in Fig. 9a–f, where it can be seen that the pH 3 led to a degradation time of 180 min, while pH 12 led to a degradation time of just 18 min, verifying the degradation of erythrosine under the highly alkaline dye solution. These findings indicate that the ZnO surface could have been deactivated *via* its instability in the acidic pH of the dye solution and lesser amount of hydroxyl radicals, as shown in Fig. 9a. However, in alkaline pH, the solution was enriched with hydroxyl radicals and the surface activation would be higher, and thus S-15 of ZnO was found to be most efficient in alkaline pH of 12, as shown in Fig. 9e. Also, the dye kinetics and degradation rate were studied in varied pH dye solutions, and an excellent pH-dependent performance towards dye decomposition could be seen, as shown in Fig. 10a and b. The rate constant for the degradation of erythrosine using 15 mg of S-15 of ZnO was found to be higher at pH 12, and also the degradation rate was higher too. The dye removal % was also studied under the influence of varying the pH using 15 mg S-15, and 100% dye removal in alkaline pH 12 was observed, as shown in Fig. 10c and Table 2. The presented pH study revealed that the local pH environment around the catalyst surface could have a significant role in oxidation of the dye. The overall photocatalytic performances of the *Moringa oleifera* leaves extract-assisted ZnO nanostructures, in terms of the initial dye concentration, catalyst dose, and pH of dye solution, are given in Table 2. The fitting data shown in Fig. 6b, c, 7d, e, 8d, e and 10a, b were treated with a first-order kinetics model. The experimental data did not fit adequately. The *R*^2^ and *K* values are given in Table 2. The *R*^2^ values were found to be between 0.94 to 0.99. In many cases, the fitting through *R*^2^ values was either 0.99 or close to 0.98, indicating the pseudo-first-order kinetics model. Therefore, the kinetics of erythrosine degradation could be more closely described by the pseudo-first-order kinetics model and exhibited relatively large rate constant (*K*) values.

**Table 2:** Brief performance evaluation of pure ZnO and green synthetized materials towards erythrosine dye degradation under different degradation parameters

Sample ID	Dye conc.	Catalyst dose	Time (min)	Degradation (%)	*R* ^2^	Rate constant (K min)
Pure ZnO	9.09 × 10^5^ M	10 mg	175	38.8	0.95	2.87 × 10^−3^
Sample-1				67.8	0.93	6.27 × 10^−3^
Sample-2				86.1	0.96	1.13 × 10^−2^
Sample-3				91.8	0.94	1.39 × 10^−2^

**Dose study**						
Sample-3	4.54 × 10^5^ M	5 mg	90	79.4	0.95	1.71 × 10^−2^
		10 mg		92.6	0.99	2.91 × 10^−2^
		15 mg		98.8	0.98	4.71 × 10^−2^

**pH study**						
Sample-3	4.54 × 10^5^ M	pH 4	180	54.9	0.94	4.75 × 10^−3^
		pH 6	120	87.4	0.98	1.75 × 10^−2^
		pH 8	60	92.9	0.99	4.52 × 10^−2^
		pH 10	30	94.3	0.99	9.24 × 10^−2^
		pH 12	18	98.5	0.98	2.20 × 10^−1^

**Fig. 11:**
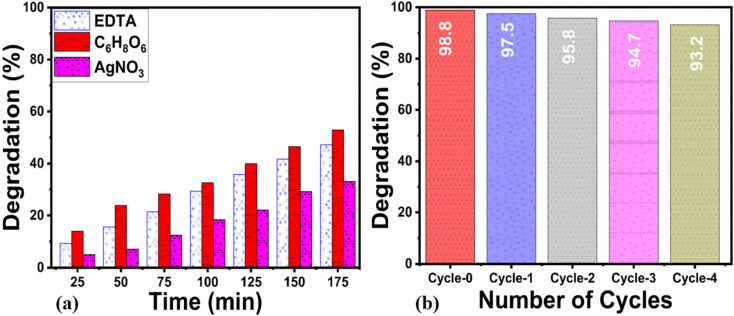
(a) Cycling stability of ZnO nanostructures synthesized with 15 mL (S-15) *Moringa oleifera* leaves extract using a catalyst dose of 15 mg, (b) scavenger analysis of ZnO nanostructures synthesized with 15 mL (S-15) *Moringa oleifera* leaves extract using a catalyst dose of 15 mg.

**Scheme 2:**
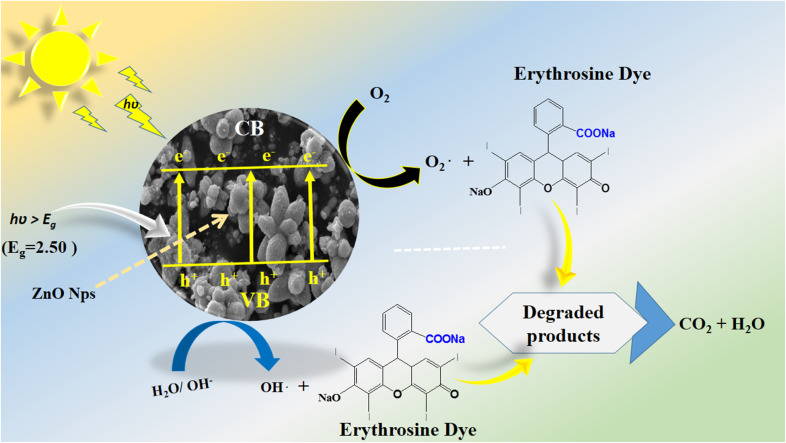
Photocatalytic oxidation mechanism of erythrosine dye on the modified ZnO nanostructures.

For assessing the long-term use of photocatalysts, the photocatalyst stability is a highly crucial factor. Here, the stability was explored by the use of a 15 mg catalyst dose of S-15 through collecting and reusing it during several cycles for the degradation of erythrosine dye. The catalyst collection was done by centrifugation, with negligible loss of material seen, which might have slightly decreased the dye removal %, as shown in Fig. 11a. The control cycle could be seen with a dye removal % of 98.8%, whereas after 4 recycling measurements, the dye removal % was around 93.2%. This indicates that the *Moringa oleifera* leaves extract could enhance the stability of ZnO, which is promising for the real dye aqueous sample analysis. The SEM image of S-15 after the cycling stability tests is shown in ESI Fig. (S1),† suggesting the morphology of ZnO was retained and that it exhibited excellent stability. For illustrating the role of the oxidizing agent towards the oxidation of erythrosine in aqueous solution, radical trapping measurements were performed using silver nitrate (AgNO_3_), ascorbic acid (C_6_H_8_O_6_), and ethylenediamine tetracetate (EDTA), as shown in Fig. 11b. The relative role of these scavengers towards the decrease in the degradation rate of erythrosine is shown in Fig. 11b. It could be seen that silver nitrate had a prominent effect in trapping the hydroxyl radicals, indicating the major role of radicals in participating in the degradation of erythrosine under natural sunlight illumination. Generally, the photocatalytic oxidation of dyes can be described by the proposed mechanism shown in Scheme 2. In this mechanism, when photons interact with the semiconducting material, it excites valence electrons to move into the conduction band, leaving holes in the valence band. Both the photogenerated electrons and holes participate in the photodegradation of dyes. To enrich the formation of hydroxyl radicals, a high surface area material is preferred as it can facilitate the adsorption of oxygen molecules on the catalytic material, thus an enhanced photocatalytic performance could be achieved. These radicals, like hydroxyl, oxide, and peroxide, are generated *via* the interaction of electron and holes with dissolved oxygen in the dye solution and water molecules, hence the dye starts to be decomposed, as shown in Scheme 2. A major consideration in designing efficient ZnO-based photocatalysts is to eliminate or minimize the recombination rate of electrons and holes, which is a challenge; however, the *Moringa oleifera* leaves extract in the present study played a vital role in improving the photocatalytic performance of ZnO through minimizing the recombination rate of electron–hole pairs.

Furthermore, the antibacterial activity of the as-synthesized ZnO samples was investigated to assess the multiple uses of ZnO-based materials. It has been shown that ZnO has improved antibacterial activity for Gram positive and Gram negative bacteria.^60^ The antibacterial activity is significantly affected by several factors, such as the size of the nanoparticles, synthetic method, susceptibility to bacterial action, and shape structure.^61^ Also, it has been found that ZnO nanoparticles prepared by green methods have improved antibacterial activity.^62^ A study was performed to investigate the use of ZnO prepared with *Moringa oleifera* leaves extract for its antibacterial activity against *E. coli* and *Bacillus subtilis* as Gram-negative and Gram-positive bacterial strains, respectively. The antibacterial activity of the *Moringa oleifera* leaves extract-assisted ZnO samples was also compared with that of pure ZnO. Their corresponding inhibition zones located in Petri dishes are shown in ESI Fig. (S2a and b).† It could be seen that S-15 of ZnO prepared with *Moringa oleifera* leaves extract exhibited a large inhibition zone towards *E. coli* and *Bacillus subtilis* pathogens. A bar graph was made for a simple representation of the antibacterial activities of pure ZnO and the *Moringa oleifera* leaves extract-assisted ZnO samples, as shown in ESI Fig. (S2c).† The enhanced antibacterial activity of S-15 could be related to production of more toxic oxygenated radicals and their rapid penetration into microorganisms. The gram negatively charged bacterial strain may bound to positively charged zinc ion which ultimately gives out death. A photocatalytic comparative study of S-15 was done with some recent reported photocatalysts,^63–75^ as shown in ESI Table (S1).† It could be seen that the proposed photocatalyst offers advantages of high efficiency, low cost, and ecofriendliness over existing photocatalytic materials.

## 4. Conclusions

The facile and ecofriendly synthesis of ZnO nanostructures was performed using *Moringa oleifera* leaves extract using a modified hydrothermal method. The SEM study showed that the surface morphology of ZnO turned from nanorods to elongated nanoparticles with a reduced size. The XRD results revealed that the two theta shift was larger for the S-15 sample of ZnO prepared with 15 mL *Moringa oleifera* leaves extract. The S-15 sample of ZnO showed 100% removal percentage of erythrosine dye in alkaline pH 12 in 18 min using a low dye concentration under natural sunlight illumination. Also, S-15 was found to be highly active in killing *E. coli* and *Bacillus subtilis* pathogens. The enhanced dual performance of S-15 could be assigned to its modified surface, reduced optical band gap, and favorable surface morphology.

## Data availability

The data and materials are available from the corresponding author upon reasonable request.

## Conflicts of interest

Authors have no conflict of interest in the presented research work.

## Supplementary Material

RA-015-D4RA08782H-s001
