# Supplementary material for: Moringa oleifera leaves extract-mediated synthesis of ZnO nanostructures for the enhanced photocatalytic oxidation of erythrosine
Source: RSC Adv. 2025 Jan 27;15(4):2810–24. doi: 10.1039/d4ra08782h (PMC11771134; doi:10.1039/d4ra08782h)
Supplement: RA-015-D4RA08782H-s001 [file RA-015-D4RA08782H-s001.pdf]

Supplementary Information (SI) for RSC Advances.
This journal is © The Royal Society of Chemistry 2025

Moringa *oleifera* leave extract mediated synthesis of ZnO nanostructures for the enhanced photocatalytic oxidation of erythrosine.

Noshaba Parven[1], Khalida Faryal Almani[1], Muhammad Ali Bhatti[2], Aneela Tahira[4], Omm-e-Hany[4], Aqeel Ahmed Shah[6], Ayman Nafady[7], Matteo Tonezzer[8], Zafar Hussain Ibupoto[3*]

[1]*Departement of Pharmaceuticals, University of Sindh, Jamshoro 76080, Pakistan.*

[2]*Centre for Environmental Sciences, University of Sindh Jamshoro, 76080, Sindh, Pakistan.*

[3]Institute of Chemistry, University of Sindh, Jamshoro 76080, Pakistan.

[4]*Institute of Chemistry, Shah Abdul Latif University KhairpurMirs, Sindh, Pakistan.*

[5]*Department of Environmental Studies, University of Karachi, 75270, Sindh, Pakistan.*

[6]Wet Chemistry Laboratory, Department of Metallurgical Engineering, NED University of Engineering and Technology, University Road, Karachi 75270, Pakistan.

[7]Chemistry Department, College of Science, King Saud University, Riyadh, 11451, Saudi Arabia.

[8]Department of Chemical and Geological Sciences, University of Cagliari, Monserrato, Italy.

[*]*Corresponding author(s): Zafar Hussain Ibupoto, PhD, Email :zaffar.ibhupoto@usindh.edu.pk*

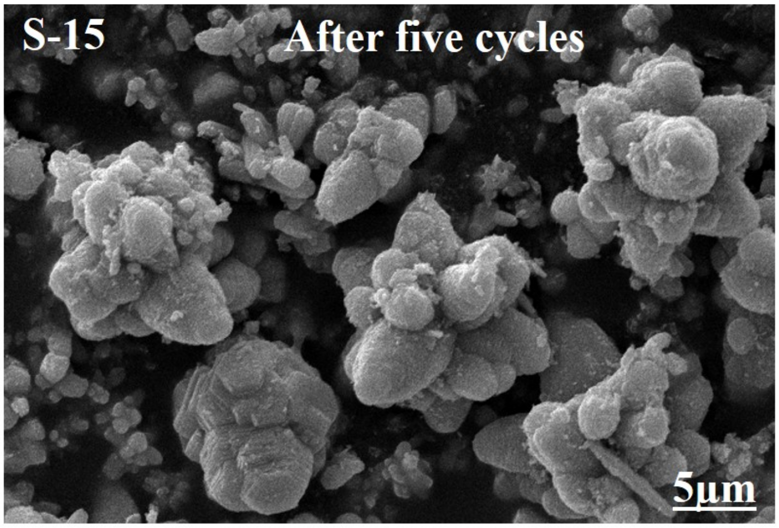

Figure (S1): SEM image of S-15 after cycling stability test

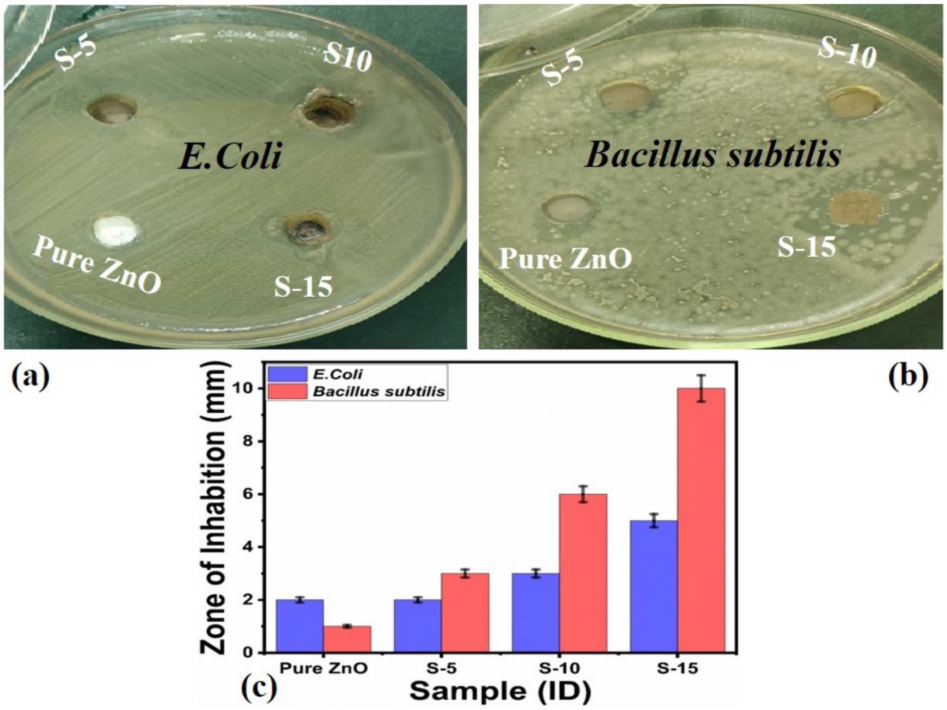

Figure (S2): (a, b) Antibacterial activity of pure ZnO and ZnO nanostructures synthesized with 5mL (S-5), 10mL(S-10) and 15 mL(S-15) of Moringa *oleifera* leaves extract respectively in E.Coli and their petri dish view with inhibition zone, (c) Corresponding measured inhibition zone in mm.

Table (S1): Comparative analysis of green assisted ZnO nanoparticles using sample 3 with pervious reported work

**Photocatalyst**	**Light source**	**Time (min)**	**Catalyst dose**	**Degradation (%)**	**Ref.**
ZnO NPs	UV-lamp	100	15 mg	99.8	Herein
ZnO	Hg-lamp 10 W	120	100 mg	90.0	63
Mn-ZnO	UV Lamp	90	24 mg	60.0	64
ZnO/AC	UV-lamp	45	0.25 g/L	92.2	65
Ag-ZnO	Solar radiation	30	100 mg	98.5	66
Ag-ZnO@GO	Xe-lamp 20 W	180	20 mg	85.0	67
ZnO/CuO	Solar radiation	120	0.5 g/L	93.0	68
ZnO/PNA	UV-lamp 250 W	120	200 mg	85.0	69
Cd-ZnS	Solar radiation	120	30 mg	96.7	70
$CuO/ZnO/TiO_2$	UV-light 6 W	180	100 mg	100	71
ZnO/GO	Xe-lamp 300 W	90	10 μg/mL	95.6	72
ZnO SCs	LED lamp 23 W	780	0.1 g	58.0	73
30%ZnO/Nylon	UV-light 15 w	300	0.01 g	58.3	74
CNDE2.0	A350 W xenon lamp	120	50 mg	90%	75

**Photocatalyst**	**Light source**	**Time (min)**	**Catalyst dose**	**Degradation (%)**	**Ref.**
**Sample-3**	**Sun light**	**90**	**15mg**	**98**	**Herein**
